# Isolation, Identification, and Genetic Evolution Analysis of *VP1* Gene of Feline Calicivirus Strain ZZ202306

**DOI:** 10.3390/ijms26062565

**Published:** 2025-03-13

**Authors:** Shi-Jun Zhang, Dan Su, Shi-Bo Zhao, Jia-You Xing, Lei Zeng, Jiang Wang, Sheng-Li Ming, Bei-Bei Chu

**Affiliations:** 1 College of Veterinary Medicine, Henan Agricultural University, Zhengzhou 450046, China; 18134405668@163.com (S.-J.Z.); 17803844064@163.com (D.S.); z1253959849@163.com (S.-B.Z.); xingjiayou123@163.com (J.-Y.X.); zenglei2021918@163.com (L.Z.); wangjiang@henau.edu.cn (J.W.); 2 Key Laboratory of Animal Biochemistry and Nutrition, Ministry of Agriculture and Rural Affairs, Zhengzhou 450046, China; 3 Key Laboratory of Animal Growth and Development of Henan Province, Henan Agricultural University, Zhengzhou 450046, China; 4 International Joint Research Center of National Animal Immunology, Henan Agricultural University, Zhengzhou 450046, China

**Keywords:** FCV, isolation and identification, biological characteristics, genetic evolution

## Abstract

This study investigated a suspected Feline calicivirus (FCV) outbreak at a veterinary facility in Zhengzhou, Henan Province, China. RT-PCR analysis confirmed the FCV presence, with subsequent CRFK cell culture propagation leading to the isolation and characterization of strain ZZ202306. Immunofluorescence and Western blot analyses validated the specificity of monoclonal antibodies targeting the FCV VP1 capsid protein. Transmission electron microscopy revealed non-enveloped virions of ~40 nm in diameter, exhibiting typical caliciviral architecture. Viral replication kinetics demonstrated exponential growth between 6 and 18 h post-inoculation, reaching a peak titer of 10^7.96^ TCID_50_/0.1 mL. Genomic sequencing coupled with phylogenetic reconstruction of the *VP1* gene revealed a close genetic relation to domestic Chinese strains and international variants, while maintaining distinct evolutionary divergence from other calicivirus genera.

## 1. Introduction

Feline calicivirus (FCV) is a member of the genus Vesivirus within the family Caliciviridae [1], commonly affecting both domestic and wild felids. The predominant clinical manifestation of FCV infection is oral ulceration, accompanied by mild respiratory symptoms. Additional clinical signs may include pneumonia, lameness, gastrointestinal disturbances, conjunctivitis, and feline chronic gingivostomatitis. FCV is prevalent in cat populations globally. In 2017, J. Hou reported that the prevalence of FCV in Europe was 22.2% [2]. From 2018 to 2020, Longlong Cao’s study on viral diseases in domestic cats revealed a 10.86% FCV positive detection rate [3]. The widespread distribution of FCV in cat populations is partly due to its transmission mode. FCV primarily spreads through contact with oral, nasal, and conjunctival tissues, and some cats may continue to shed the virus post-recovery, enhancing its transmissibility and complicating disease control [4].

FCV is a non-enveloped, single-stranded, positive-sense RNA virus with a particle diameter ranging from 35 to 39 nanometers. Its capsid consists of 32 intermediate concave cup-shaped subunits arranged in T = 3 icosahedral symmetry, comprising a total of 90 capsomeres [5]. The FCV genome, approximately 7.7 kilobases in length, contains three open reading frames (ORFs). ORF2, approximately 2.0 kilobases in length, encodes the viral capsid protein. Initially, ORF2 translates a precursor capsid protein of approximately 75 kDa, which is subsequently processed by a viral protease to remove 124 amino acids from the N-terminus, resulting in the mature 62 kDa *VP1* capsid protein [6].

FCV, first isolated by Fastier in 1957 from a domestic cat in New Zealand, has been detected in various feline species, including lions, tigers, and cheetahs, highlighting its global distribution across Europe, the Americas, and Asia [7,8]. In 2009, Di Martino and colleagues isolated FCV from a puppy, suggesting potential cross-species transmission [9]. FCV is highly contagious among cats, causing respiratory disease characterized by oral ulcers, fever, sneezing, rhinitis, and conjunctivitis, which complicates disease control efforts. Clinically asymptomatic cats can shed the virus for prolonged periods, leading to high morbidity but low mortality [10,11,12]. Vaccination, notably targeting prevalent strains such as 255 and F9, remains the primary preventive measure against FCV. However, as an RNA virus, FCV is prone to mutations that can reduce vaccine efficacy, posing significant threats to the health and survival of domestic and wild felids [3].

In 2023, ocular, nasal, and pharyngeal swabs were collected from a cat suspected of FCV infection at a veterinary hospital in Zhengzhou. These samples underwent RT-PCR testing and viral isolation, followed by the characterization of the isolated FCV strain through viral quantification and *VP1* gene sequencing. The primary objective of this study is to elucidate the mutation patterns of FCV in China, providing a scientific basis for the development of domestically produced vaccines. Additionally, this research aims to establish a reference framework for researchers, veterinarians, and cat caregivers to implement effective FCV control strategies and guide future research directions. Ultimately, our goal is to contribute to the development of FCV vaccines, thereby reducing the virus’s impact on feline populations and their human companions.

## 2. Results

### 2.1. Identification of Pathogens in Sample

**Figure 1:**
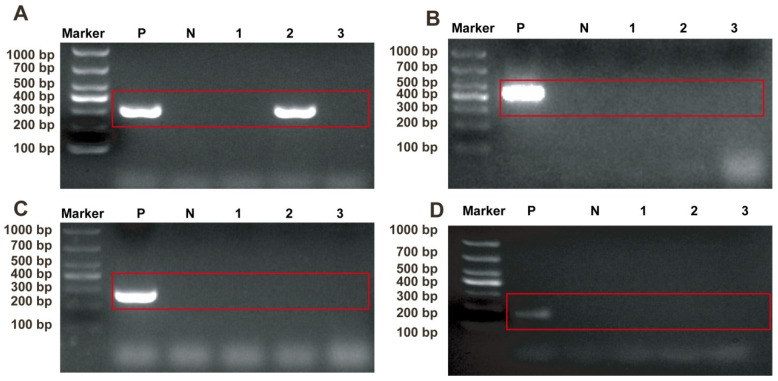
Identification of clinical samples with pathological characteristics. (**A**) Detection of FCV in tissue sample. (**B**) Detection of FPV in tissue sample. (**C**) Detection of FHV in tissue sample. (**D**) Detection of FCoV in tissue sample. P: Positive control; N: negative control; and 1–3: test samples.

The samples were analyzed using PCR with specific primers targeting common feline pathogens: FCV, FPV, FHV, and FCoV. The agarose gel electrophoresis of the amplified nucleic acids revealed a distinct band at 321 bp, indicative of FCV presence in one sample (Figure 1A). No positive bands were observed for FPV (Figure 1B), FHV (Figure 1C), or FCoV (Figure 1D), providing initial evidence of the presence of FCV and the absence of the other three feline viruses in the sample.

### 2.2. Isolation and Identification of FCV

**Figure 2:**
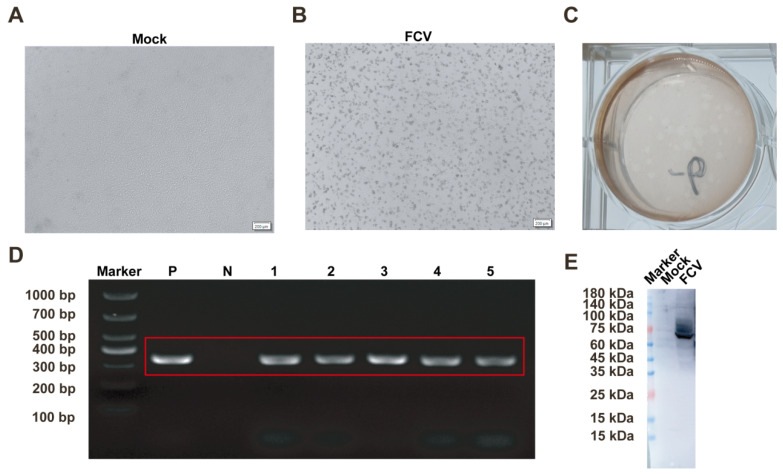
Isolation and Identification of FCV. (**A**) Morphology of uninfected CRFK cells. (**B**) Morphology of CRFK cells post-FCV infection. (**C**) PCR detection of FCV in plaque-purified samples following serial passages. (**D**) PCR detection of samples after serial passages. (**E**) Western blotting identification of FCV. Scale bar: 200 μm; 1–5: virus supernatants from each of the five serial passages; P: positive control; and N: negative control.

The processed tissue samples were inoculated onto CRFK cells. FCV infection resulted in cellular shrinkage, detachment, and a grape-like cytopathic effect under optical microscopy (Figure 2A,B). The plaque purification of FCV was performed, with plaques remaining visible after three rounds (Figure 2C). The continuous passaging of the virus on CRFK cells consistently demonstrated cytopathic effects across five passages. PCR analysis of supernatants from each passage consistently detected FCV-specific bands (Figure 2D). Following infection, viral proteins were extracted and identified via Western blotting, confirming the isolate as FCV based on recognition of the VP1 protein by the positive serum (Figure 2E).

### 2.3. Detection of FCV Using Immunofluorescence Assay and Electron Microscopy

**Figure 3:**
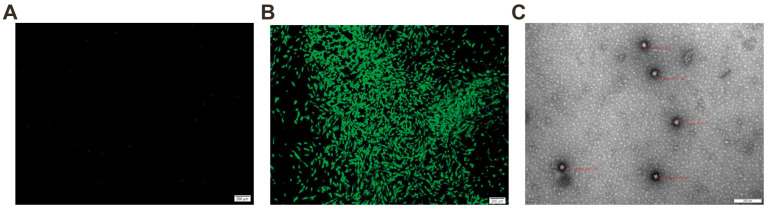
Characterization of FCV strain ZZ202306. (**A**) Uninfected CRFK cell. (**B**) CRFK cell post-FCV infection. (**C**) Electron microscopic imaging of supernatants from FCV-ZZ202306-inoculated CRFK cells using negative staining with phosphotungstic acid.

An immunofluorescence assay was conducted on FCV-infected CRFK cells using a specific mouse monoclonal antibody targeting the FCV VP1 protein, with normal CRFK cells serving as controls. The assay revealed a specific immunofluorescence in FCV-infected CRFK cells (Figure 3A), while normal CRFK cells exhibited no fluorescence (Figure 3B). The negative staining and electron microscopy of the F5 generation virus supernatant from the FCV isolate showed non-enveloped, spherical virus particles with a diameter of approximately 40 nanometers (Figure 3C). These observations confirm the isolated strain as FCV STRIAN ZZ202306 in this study.

### 2.4. Viral Titer Determination for FCV Strain ZZ202306

**Figure 4:**
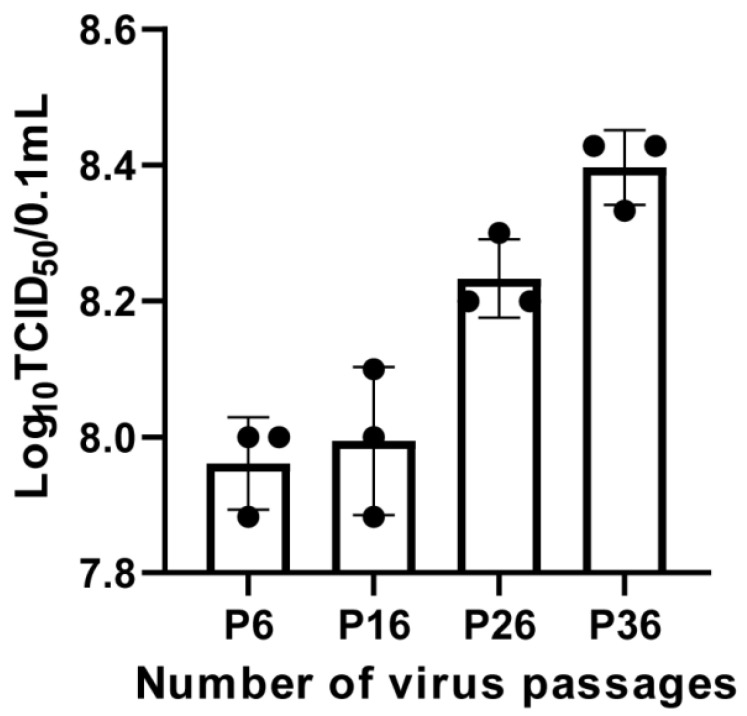
Viral titer determination of FCV strain ZZ202306.

The FCV strain ZZ202306 was continuously passaged on CRFK cells, and viruses from the 6th, 16th, 26th, and 36th generations were subjected to tenfold serial dilution and the subsequent infection of the CRFK cells. After a seven-day incubation period, the TCID_50_ of the isolated virus was calculated using the Reed–Muench method, resulting in TCID_50_ values of 10^7.96^/0.1 mL, 10^7.99^/0.1 mL, 10^8.23^/0.1 mL, and 10^8.40^/0.1 mL for the 6th, 16th, 26th, and 36th generation viruses, respectively (Figure 4).

### 2.5. Proliferation Dynamics of FCV Strain ZZ202306 in CRFK Cells

**Figure 5:**
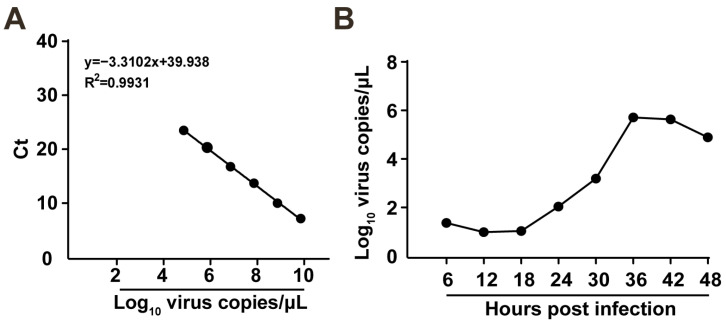
Proliferation kinetics of FCV strain ZZ202306. (**A**) Standard curve of FCV-VP1 recombinant plasmid. (**B**) One-step growth curve of FCV strain ZZ202306.

The replication kinetics of the FCV strain ZZ202306 isolate in CRFK cells were investigated by inoculating the virus at a multiplicity of infection of 0.01. Virus samples were collected at 6, 12, 18, 24, 30, 36, 42, and 48 h post-infection for viral RNA extraction and quantitative real-time PCR analysis. The assay demonstrated a strong linear correlation within a dilution range from 10^−2^ to 10^−8^, with a linear equation of y = −3.4814x + 41.594 (R^2^ = 0.9994) (Figure 5A), establishing a standard curve. The data indicated that the isolate exhibited its highest replication rate between 18 and 36 h, reaching peak viral copy numbers at 36 h post-infection (Figure 5B).

### 2.6. Amino Acid Homology and Phylogenetic Analysis of FCV Strain ZZ202306-VP1Gene

**Figure 6:**
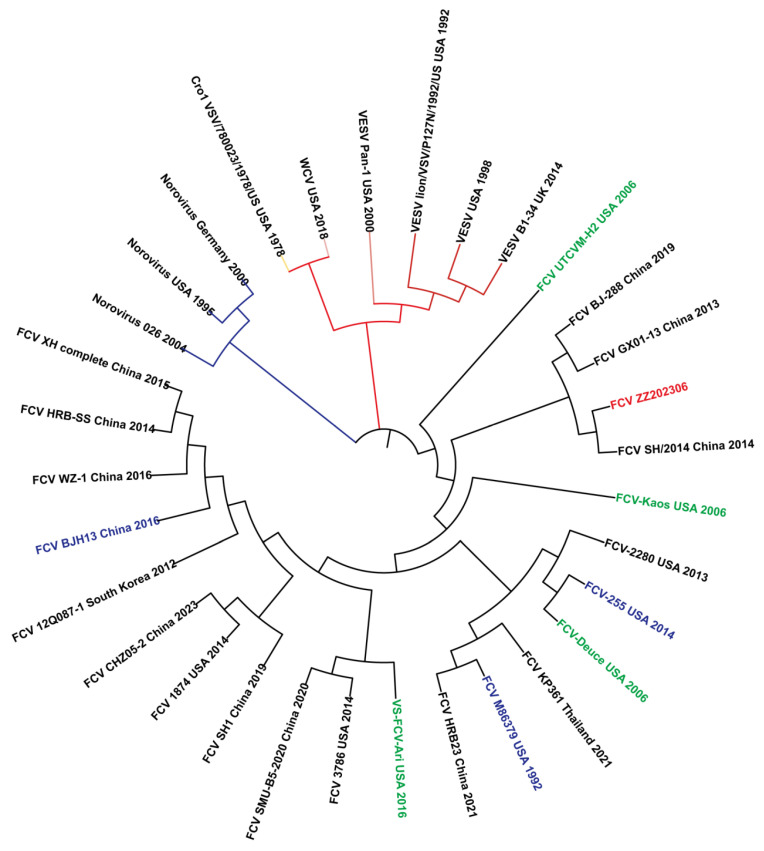
Nucleotide homology analysis of FCV strain ZZ202306 *VP1* gene.

The nucleotide sequence of the FCV strain ZZ202306 *VP1* gene was analyzed for homology with domestic and international isolates, revealing a fragment length of approximately 2010 base pairs. A comparison of the *VP1* gene sequences between the FCV strain ZZ202306 (isolated in 2023) and other FCV reference strains was performed using MegAlign software in DNAStar Lasergene v7.1 and MEGA7 software to construct an amino acid phylogenetic tree (Figure 6). The amino acid sequence of the *VP1* gene of the FCV ZZ202306 isolate exhibited 83.6% to 87.2% identity with various domestic and international reference strains, including 85.8% identity with the domestic vaccine strain 255. In contrast, the homology with caliciviruses from other species ranged from 15.6% to 41.6%. A phylogenetic analysis placed the FCV strain ZZ202306 and the FCV strain SH/2014 (2014) within the same evolutionary branch, while the 255 vaccine strain formed a distinct branch, and caliciviruses from other species were placed on a separate, more distant branch.

### 2.7. Analysis of Amino Acid Mutations in Highly Mutated Region of FCV Strain ZZ202306-VP1 Gene

**Figure 7:**
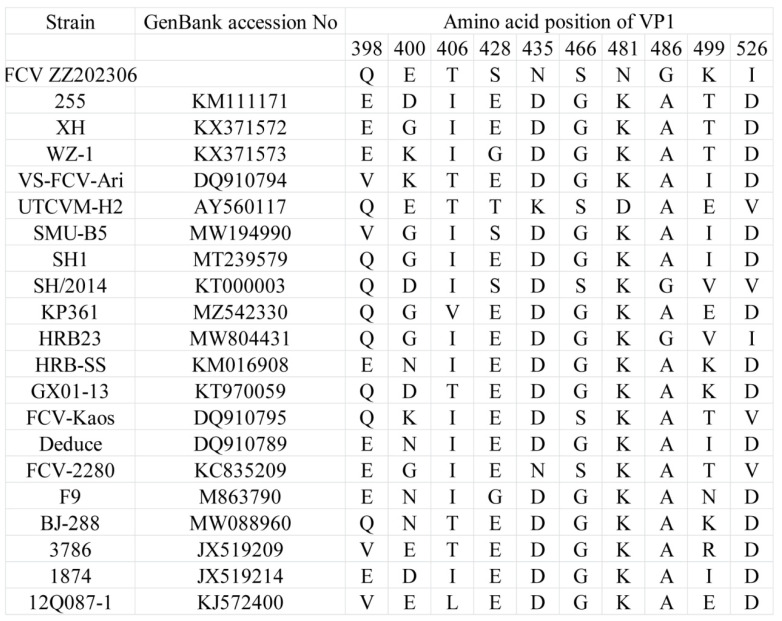
Amino acid mutations in hypervariable region of FCV strain ZZ202306 VP1.

Regions C and E within the open reading frame 2 (ORF2) of the FCV *VP1* gene are recognized as hypervariable among the six regions (A to F) [13]. In structural biology, the VP1 protein is organized into the N-terminal arm region, the shell region, and the protruding region, which is further divided into the proximal P1 and the distal P2 regions [14,15,16]. The P2 subdomain, located within the hypervariable region, contains FCV neutralizing epitopes [17,18,19]. Amino acid analysis of the FCV strain ZZ202306 identified 10 mutations in the hypervariable region. Protein structure predictions indicated that the mutations E400D, T406I, S428E, N435D, N481K, G486A, K499T, and I526D are located on the surface-exposed loops of the P2 region, while the S466G and G486A mutations are internally positioned or less accessible from the capsid surface. Notably, the T406I mutation may distinguish classical strains (F9, 255, and WZ-1) from highly pathogenic strains of FCV (VS-FCV-Ari and UTCVM-H2). In the FCV strain ZZ202306, the amino acid at position 406 is threonine (Figure 7).

## 3. Discussion

In this study, a FCV infection was investigated, leading to the successful isolation and characterization of a novel strain designated FCV strain ZZ202306. The biological analysis of this isolate revealed a viral titer of 10^7.9^ TCID_50_/0.1 mL, which is lower than that of the previously isolated domestic strains, such as those from the Shanghai region in 2021, which exhibited titers of 10^8.73^ and 10^10.26^ TCID_50_/mL. In Beijing, Zhonghua Zhang isolated five strains with titers ranging from 10^8.6^ to 10^7.3^ TCID_50_/mL [20], suggesting that the FCV strain ZZ202306 aligns more closely with classical FCV strains. Additionally, the amino acid identity between the isolate and the domestic vaccine strain 255 is 85.8%, and with other classical FCV strains, it ranges from 83.6% to 87.2%. Consistent with Liu Jian’s findings, the *VP1* nucleotide sequence similarity among 13 FCV isolates in Shanghai ranged from 74.3% to 99.8%. The amino acid similarity with domestic and international reference strains was between 82.7% and 91.9%, and 82.7% and 89.4% with the vaccine strains [21]. The *VP1* nucleotide and amino acid sequence similarities among the isolates are relatively low, with mutations predominantly occurring in high-mutation regions, aligning with the known mutation characteristics of FCV [22].

Phylogenetic analysis indicates that the isolate and the virulent strain SH2014 belong to the same evolutionary clade, suggesting minimal genetic divergence in the *VP1* gene, potentially due to a recent common ancestor or similar evolutionary trajectories. Conversely, the isolate and the domestic vaccine strain 255 are located on distinct evolutionary branches, indicating significant genetic divergence, which may imply that the isolate has accrued sufficient genetic alterations in the *VP1* gene that could impact viral antigenicity or host interactions [23]. The isolate also forms a more distant clade with caliciviruses from other species, suggesting a unique evolutionary pathway for FCV in the *VP1* gene or limited genetic exchange with other calicivirus groups.

Given the low fidelity of RNA polymerase during replication, FCV is highly susceptible to mutations. Furthermore, the application of vaccines and natural immune responses exert selective pressures on FCV, prompting evolutionary adaptations to evade immune recognition and clearance [13,24,25]. This selective pressure can lead to antigenic shifts within the virus population, potentially resulting in the emergence of new strains [26]. For the FCV-ZZ202306 strain, its cellular virulence tends to resemble that of classical strains [13]. However, the comparative analysis of amino acid sequences revealed that seven amino acid residues (positions 430, 438, 443, 448, 452, 455, and 458) in region E may be linked to its pathogenicity. This study identified five amino acid sites in the isolate that correspond to the previously reported VS-FCV strain, indicating that the FCV strain ZZ202306 has the potential to evolve into a virulent strain, thereby complicating FCV prevention and control efforts. Additionally, research has demonstrated that the P2 region of the FCV VP1 protein serves as the virus’s receptor-binding domain [17,27,28], and the 10 amino acid mutations in this isolate may facilitate interspecies transmission.

In conclusion, we have successfully isolated and characterized a new FCV strain, ZZ202306, providing insights into the genetic variability of the *VP1* gene. This research underscores the importance of conducting cross-immunoprotection assays between prevalent local strains and vaccines, emphasizing the critical need for the evaluation of vaccine efficacy to develop more effective vaccines. This approach is anticipated to reduce FCV infection rates and associated mortality.

## 4. Materials and Methods

### 4.1. Cell Culture and Reagents

The CRFK feline kidney cell line was maintained in our laboratory using cell culture supplies from GIBCO, Miami, FL, USA, including Minimum Essential Medium (MEM) and fetal bovine serum. The DNA/RNA extraction kit and gel extraction kit were purchased from TIANGEN Biotech (Beijing) Co., Ltd., Beijing, China. The All-in-one First-strand Synthesis MasterMix was purchased from Zhengzhou Xinzhiyi Biotechnology Co., Ltd., Zhengzhou, China. DNA markers DL2000 and DL5000, and PrimeSTAR Max Premix DNA, were purchased from TaKaRa Biotechnology (Dalian) Co., Ltd., Dalian, China.

### 4.2. Sample Processing

Oral and nasal secretions were collected from sick cats suspected of FCV infection at Zhengzhou Pet Hospital, which were then mixed with approximately 1 mL of phosphate buffered saline (PBS). The mixture was vortexed to achieve homogeneity, followed by the addition of 3% antibiotics and incubation overnight at 4 °C. After incubation, samples were centrifuged at 8000× *g* for 5 min, and the supernatant was carefully collected. Sterilization was performed by filtering the supernatant through a 0.22 μm membrane filter. Filtered samples were stored at −80 °C for subsequent analysis.

### 4.3. Specimen Testing

Primers for FCV, feline parvovirus (FPV), feline herpesvirus (FHV), feline coronavirus (FCoV), and FCV-specific identification were synthesized according to literature references [29]. Full-length amplification primers for the FCV *VP1* gene were designed based on the gene sequence of the FCV-F9 standard strain sequence (GenBank accession No. M863790). Primer sequences are detailed in Table 1 and were synthesized by Sangon Biotech (Shanghai) Co., Ltd., Shanghai, China. DNA/RNA extraction from samples was performed using the DNA/RNA extraction kit, followed by reverse transcription of RNA into cDNA using a reverse transcription kit. PCR amplification was conducted using cDNA and DNA as templates. Amplified products were detected using agarose gel electrophoresis. FCV VP1-positive PCR products were recovered using a gel extraction kit, ligated into the pET28a(+) vector, and transformed into TOP10 competent cells. Colony PCR was performed, and three positive recombinant plasmids were selected and sent to Sangon Biotech (Shanghai) Co., Ltd. for sequencing. The FCV *VP1* gene sequencing results were analyzed for homology with published vaccine strains and domestic and international prevalent strains (reference sequences in Table 2) using MegAlign 7.0, followed by construction of a gene phylogenetic tree.

### 4.4. Virus Isolation and Cultivation

PCR-confirmed FCV-positive specimens were inoculated onto CRFK cells at approximately 60% confluency. Cultures were incubated at 37 °C in a 5% CO_2_ atmosphere, and cytopathic effects (CPEs) were monitored daily. If no CPEs were observed, the supernatant was collected, subjected to repeated freeze–thaw cycles at −80 °C, and passaged onto fresh CRFK cells for further incubation. Virus passages were PCR-tested, and samples without CPEs by the fifth passage were discarded. Specimens inducing cellular deformation (net-like appearance, rounding, and detachment) and testing PCR-positive underwent plaque purification. The virus was harvested when stable CPEs were consistently observed after serial passages on CRFK cells.

### 4.5. Western Blotting

CRFK cells infected with the test isolate were sampled when CPEs appeared but before cell detachment, with a negative control concurrently set up. Fifty microliters of cell lysis buffer were added to the samples, followed by repeated pipetting until clarification. Following centrifugation at 12,000× *g* for 5 min at 4 °C, the supernatant was harvested. SDS-PAGE gel electrophoresis was performed on the viral protein samples, followed by transfer to a cellulose membrane. The membrane was blocked with 5% skim milk at room temperature for 1 h, washed with TBST, and incubated overnight at 4 °C with a monoclonal antibody against the FCV VP1 protein (prepared in our laboratory). After washing with TBST, the membrane was incubated at room temperature for 1 h with HRP-conjugated goat anti-mouse IgG (1:5000). The membrane was then photographed for observation.

### 4.6. Immunofluorescence Assay

CRFK cells were digested and seeded into a 24-well plate. At approximately 60% confluence, the test isolate was inoculated into the cells and cultured at 37 °C with 5% CO_2_. After 36 h of infection, the culture medium was discarded, and each well was fixed with 200 μL of 4% paraformaldehyde at room temperature for 30 min. Wells were washed three times with PBS, then treated with 200 μL of 0.1% Triton X-100 for 5 min at room temperature to permeabilize cells. After another PBS wash, 200 μL of 10% FBS in PBS was added to each well for blocking at room temperature for 1 h. Wells were washed with PBS, and cells were incubated overnight at 4 °C with the FCV monoclonal antibody. Wells were washed three times with PBS, incubated with FITC-labeled goat anti-mouse IgG (1:1000) for 1 h at room temperature, washed with PBS, and observed under a fluorescence microscope(Zeiss, Oberkochen, Germany).

### 4.7. TCID_50_ Assay

CRFK cells were seeded into a 96-well plate at a density of 1 × 10^5^ cells per well. At approximately 40% confluence, the test virus solution was subjected to 10-fold serial dilutions using MEM as the diluent. Each dilution was transferred to the 96-well plate, with 100 μL inoculated per well. An equal volume of MEM was used as a blank control. Plates were incubated at 37 °C with 5% CO_2_ for 3–6 days. The number of cytopathic wells was recorded, and the TCID_50_ of the virus was calculated using the Reed–Muench method.

### 4.8. Viral Growth Kinetics Analysis

The pET28a-FCV-VP1 recombinant standard plasmid was serially diluted 10-fold and used as a template for real-time fluorescent quantitative PCR. The results were analyzed by plotting the logarithm of the template copy number against the cycle threshold values to generate a standard curve. The correlation coefficient was calculated to establish a standard curve for detecting the FCV *VP1* gene. CRFK monolayer cells were infected with the virus at a multiplicity of infection of 0.01. After 1 h of adsorption, cells were washed with serum-free MEM and incubated with 1% maintenance medium at 37 °C in a 5% CO_2_ environment. At 6, 12, 18, 24, 30, 36, 42, and 48 h post-infection, virus samples were gathered and subjected to extraction of viral nucleic acids for real-time fluorescent quantitative analysis.

### 4.9. Electron Microscopy

The F5 generation cell culture of the test isolate was applied to a copper grid with a supporting membrane and stained with 1% phosphotungstic acid. Excess stain was blotted with filter paper, and the grid was examined under a transmission electron microscope.

### 4.10. Statistics

All data were analyzed in GraphPad Prism 8 software and a two-tailed Student’s t-test. *p* < 0.05 was considered statistically significant. Data are shown as the mean ± standard deviation for three independent experiments.

### 4.11. Statement of Informed Consent

The owner of the animal agreed in writing for the animal ’s test sample to be involved in the study.

## 5. Conclusions

We isolated and characterized the novel FCV strain ZZ202306, shedding light on the genetic diversity of the *VP1* gene. This study highlights the significance of performing cross-immunoprotection assays between circulating regional strains and vaccines, stressing the essential evaluation of vaccine efficacy for enhanced vaccine development. This strategy is expected to lower FCV infection rates and related mortality.

## Figures and Tables

**Table 1:** Primer sequences.

Primer Name	Primer Sequence 5’-3’	Fragment Size (bp)
09-FCoV-P205	GGCAACCCGATGTTTAAAACTGG	214
09-FCoV-P211	CACTAGATCCAGACGTTAGCTC	
56-FCV-F	TGTACTTTGCGGGACTTGGT	401
56-FCV-R	ACATTACCCACAGCTTGTGCT	
57-FHV-F	GACGTGGTGAATTATCAGC	288
57-FHV-R	CAACTAGATTTCCACCAGGA	
58-FCV-F	CAARGGAGAAAATTCDGACGA	321
58-FCV-R	GTATTTWAGCACGTTAGCGCAGGT	
85-FCV-F	cagcaaatgggtcgcggatccTGCTCAACCTGCGCTAAC	2010
85-FCV-R	ggtggtggtggtggtgctcgagTAATTTAGTCATTGAACTCC	

**Table 2:** FCV *VP1* reference sequences.

Number	Strain	GenBank Accession No.	Isolation Location	Isolation Time
1	SMU-B5	MW194990	CHINA	2020
2	HRB23	MW804431	CHINA	2020
3	SH1	MT239579	CHINA	2019
4	BJ-288	MW088960	CHINA	2019
5	WZ-1	KX371573	CHINA	2016
6	XH	KX371572	CHINA	2015
7	HRB-SS	KM016908	CHINA	2014
8	SH/2014	KT000003	CHINA	2014
9	GX01-13	KT970059	CHINA	2013
10	CH-JL3	KJ495730	CHINA	2013
11	KP361	MZ542330	Thailand	2021
12	12Q087-1	KJ572400	Korea	2012
13	12Q087-5	KJ572401	Korea	2012
14	FCV-2280	KC835209	USA(VSD)	2013
15	FCV-Kaos	DQ910795	USA(VSD)	2006
16	3786	JX519209	USA	1996
17	1874	JX519214	USA	1996
18	20879	JX519211	USA	1996
19	255	KM111171	USA	2014
20	F9	M863790	UK	1992
21	5789	JX519210	USA	2014
22	UTCVM-H2	AY560117	USA	2006
23	VS-FCV-Ari	DQ910794	USA	2007
24	Deduce	DQ910789	USA	2016

## Data Availability

The data that support the findings of this study are directly available in Mendeley Data, V2, doi:10.17632/r4zhg5hnxg.2. Research Data at: https://data.mendeley.com/datasets/r4zhg5hnxg/2 (Published: 8 February 2025).
